# A qualitative exploration of menstrual management practices and the impact of menstruation on the livelihoods of female sex workers in a peri-urban area of western Kenya

**DOI:** 10.3389/frph.2026.1817624

**Published:** 2026-06-05

**Authors:** Elish McCullough, Garazi Zulaika, Edyth A. Osire, Cynthia Akinyi, Fredrick O. Otieno, Penelope A. Phillips-Howard, Supriya D. Mehta, Linda Mason

**Affiliations:** 1 Department of Clinical Sciences, Liverpool School of Tropical Medicine, Liverpool, United Kingdom; 2 Nyanza Reproductive Health Society, Kisumu, Kenya; 3 Division of Infectious Disease Medicine, Rush University College of Medicine, Chicago, IL, United States

**Keywords:** female sex workers, intravaginal practices, Kenya, male clients, menstrual hygiene, menstruation, menstrual cup, economic livelihood

## Abstract

**Background:**

Little is known about female sex worker (FSW) experiences with menstruation and its impact on their economic livelihood and health. To support a future intervention aimed at improving menstrual hygiene management (MHM) for FSW, this study sought to explore FSW's views of MHM, its impact on work, and related practices during client engagement.

**Methods:**

This qualitative study conducted seven focus group discussions among 57 FSWs in Kisumu, western Kenya, between June and September 2023. Semi-structured guides assessed current MHM practices, the impact of menstruation on work and livelihood, and clients' perceptions of sex during menses. The data were analysed using thematic analysis.

**Results:**

Outside of sex work, FSWs primarily reported using disposable sanitary pads, with tampons, reusable pads, cloths, cotton wool and tissue also reported as methods used to absorb menstrual flow. Many FSWs described continuing sex work during menstruation out of financial necessity. Needing to hide menstrual blood flow when engaging in sex work was customary and women resorted to unsafe practices, including intravaginal cleansing or inserting cotton or tissue high into the vagina to stem bleeding. Most FSWs spoke of losing income during their menses from stopping work entirely or reducing the number of clients seen. Women reported a range of client attitudes towards sex during menstruation, from indifference to disgust, with most reporting negative views. FSWs discussed that clients frequently refused services if they became aware of menstruation and that non-payment or physical abuse from clients could occur upon discovering menstruation.

**Conclusions:**

The use of harmful practices to conceal menstrual blood during sex work underscores a critical need for safer menstrual management solutions for this population that can be used discreetly and comfortably during sex, such as menstrual discs that can be worn during sex. Negative perceptions of menstruation contribute to increased financial instability in this population.

**Clinical Trial Registration**: Clinicaltrials.gov NCT05666778; Pan African Clinical Trials Registry 202305912778108.

## Introduction

In Kenya, the Ministry of Health estimated there were approximately 168,000 female sex workers (FSWs) in the country as of 2018 (1). FSWs bear an increased risk of sexually transmitted infections (STIs) and HIV (2). While the HIV prevalence is 6.2% among Kenyan women in the general population, it is 29.3% among women who engage in sex for their livelihood (3). Sex workers face heightened social and service discrimination, alienation from friends and family, exposure to violence, and financial hardship (4–7). In a study of Kenyan FSWs in Nairobi, 33.9% had experienced hunger in the past week, 19.5% reported housing insecurity, and 84.6% reported having two or more people dependent on their income (7). This economic precarity leads to many FSWs needing to continue sex during menstruation (8–13), with some engaging in harmful menstrual health management (MHM) practices to hide their bleeding (8–10, 12, 13). However, the ramifications of poor menstrual health and hygiene (MHH) among FSWs are poorly researched, with few studies examining their unique needs while working and engaging with clients.

While common among FSWs (11, 13, 14), vaginal sex during menstruation carries added risks. Clients may have negative perceptions of sex with women who are menstruating, believing it to be “dirty”, “risky”, and “unpleasurable” (15). Consequently, some women report resorting to unsafe MHM practices, including intravaginal cleansing or intravaginal insertion of cotton to hide any bleeding from their clients. One study in Brazil among 1,000 FSWs (10) reported that 42% of FSWs used “cotton and other substances” (such as “sponge” and “mattress stuffing”) to hide menstruation, practices qualitatively reported to be associated with bad odour, discharge, pain during intercourse and chronic vaginal infections (10). A 2008 qualitative study of 30 FSWs in Mozambique similarly reported women washed internally and/or inserted sponges into their vaginas to conceal menstruation from clients (8). A 2008–2009 study of women employed in guest houses across Uganda and Tanzania found that the frequency of daily intravaginal cleansing increased during menstruation (4.7 mean cleansing acts/day in Uganda and 4.0 in Tanzania) compared to when not menstruating (4.5 mean cleansing acts/day in Uganda and 3.7 in Tanzania) (13). During in-depth interviews, women reported using “herbs or commercial substances to stop the flow of menses before transactional sex, as men were unwilling to have sex with menstruating women” (12), while a second study reported women used methods like drinking lemon juice, squeezing lemon juice into the vagina, or taking medication to temporarily stop menstrual flow (9).

Meta-analyses have shown that practices including cleansing with soap, inserting paper or cloth, and using tightening or drying agents increase the risk of HIV and/or bacterial vaginosis (BV) (16, 17). BV in turn increases the risk of conditions including sexually transmitted infections, pelvic inflammatory disease, miscarriage and preterm birth (18–26). These adverse health outcomes are hypothesised to arise from physical abrasions to the vaginal epithelium or disruption of normal protective vaginal flora caused by IVPs (16, 17).

In low- and middle-income countries (LMICs), commercial disposable menstrual pads are expensive and often unavailable, forcing women and girls to resort to substandard items (27), and engage in transactional sex for products (28). The use of cheaper reusable pads is hampered by challenges with cleaning them and the taboos surrounding open-air drying (29, 30). The use of traditional materials such as cotton wool, socks, grass, cloths, rags and paper for MHM is commonly reported (28, 31). Furthermore, poor MHM is associated with urinary tract infections (UTIs), BV (32–35), a non-optimal vaginal microbiome and STIs (36–39). Large-scale cluster-randomised control trials in western Kenya have demonstrated that adolescent girls using menstrual cups to manage bleeding were more likely to have an optimal vaginal microbiome and that cups reduced the occurrence of BV and STIs (including *N. gonorrhoea, C. trachomatis, T. vaginalis*, and HSV-2) (37–39), suggesting that menstrual cups may have beneficial effects for menstruators' sexual and reproductive health.

The present study aimed to explore current MHM practices among FSWs residing in Kisumu, western Kenya, focusing on practices used during sex work. It also sought to explore how menstruation impacts participants' client relationships and economic livelihood and their accounts of men's perceptions of sex during menses.

## Methods

### Study design

This qualitative study analysed data collected from seven focus groups among FSWs participating in the POWWeR Health Study (Periods: Optimizing Working Women's Reproductive Health Study) (40). POWWeR is a single-arm trial to assess the preliminary efficacy signal of reusable menstrual discs on non-optimal vaginal microbiomes, BV, and STIs. POWWeR will also evaluate the safety profile of the intervention and understand implementation needs. A total of 407 participants aged 15–35 were recruited by Female Peers from organisations based in Kisumu, including Kisumu Sex Workers Alliance and Keeping Alive Society's Hope, which support women who engage in transactional sex for livelihood, which has been described previously (40, 41). Participants were eligible if they were female, aged 15–35 years, exchanged sex for livelihood, and were menstruating. Those currently pregnant or having delivered within 6 months, and those using intrauterine devices (IUDs) were excluded.

Kisumu County is one of Kenya's 47 counties and has an estimated population of 1.16 million (42). It is situated on the shores of Lake Victoria and is approximately 320 km northwest of the capital, Nairobi (42, 43). The County comprises both rural and urban areas, with the Luo being the predominant tribe (42). In the 2017–2018 nationwide assessment, Kisumu County was estimated to have 5,151 FSWs, with 438 hotspots identified (1). Sexual and reproductive health in Kisumu County is of significant concern; HIV prevalence in the general adult population in 2017 was 16.3% (1).

### Focus group discussion recruitment

Women were informed about the qualitative study as part of the enrolment process and reminded at the end of the baseline visit. After approximately every 50 baseline visits were completed, a simple random sample of 12 participants was drawn for invitation to take part in the FGDs. The composition of the focus groups was designed to reflect the age distribution and work locations (e.g., guest house, street) within the study population. FGDs were conducted with enrolled FSWs before the intervention took place to assess pre-intervention MHM practices. Five FGDs were initially planned, but two additional sessions were conducted to achieve saturation of participant experiences.

### Focus group discussion approach

The study aimed to conduct five FGDs, recruiting 6–8 FSWs per focus group. Each FGD was designed to last for approximately 1.5 h. Participants received 500KES (∼$3.8) as reimbursement for transport and time. A semi-structured topic guide was developed to discuss key topics determined by the study objectives, with probes to gain further knowledge. The guide topics focused on knowledge of menstruation, current use of absorbents, hygiene practices, the impact of menstruation on livelihood, client knowledge of menstruation, differences in client knowledge across different demographic groups and client perceptions of sex during menses (see Additional File 1). The guide was refined with input from peer champions and moderators to ensure cultural relevance and clarity.

Before starting, participants provided written informed consent to participate in the FGDs, separate from the main study consent, and consent for audio recording. Participants were briefed on “ground rules,” agreeing to keep the information discussed confidential and respectful and not share it outside of the FGD. Moderators emphasised that there were no right or wrong answers and encouraged all participants to share their perspectives. To ensure confidentiality, participants were assigned numeric codes, and personal identifiers were not used throughout the FGD. The discussions were conducted in the participants' language choice of Kiswahili, Luo, or English. The female Project Coordinator (EO) served as the moderator, assisted by a female note-taker (CA). All female research staff were trained and experienced in working with FSW communities, which helped foster a safe and non-judgmental environment. The moderators used neutral, non-directive facilitation techniques to reduce social desirability bias and avoid influencing responses. No prior personal relationships existed between moderators and participants.

FGDs were digitally recorded and transcribed verbatim using a local transcription service. Transcripts were checked for accuracy while listening to the recordings. Any Luo and Kiswahili transcripts were translated into English, with a sample back-translated to check for consistency. All transcripts were de-identified, and participants were represented using the respondent number (R1 to R10) assigned to them during the focus group.

### Analysis

The analysis was conducted using a thematic framework approach chosen due to the absence of preconceived theories and because it offers a flexible approach suitable for the complexity and diversity of the data (44–46). The transcripts were imported into NVivo version 12 and read and re-read alongside the notes made during the FGDs to gain familiarity with the data. While overarching themes were pre-specified based on the study objectives, sub-themes within each domain were identified inductively from the participants' responses through iterative coding. An initial coding framework was developed, and relevant data from each transcript was coded using an iterative process, whereby new codes were added as they emerged and any text uncoded and reassigned as appropriate. The codes were then assigned under relevant themes and subthemes. Themes, subthemes, and codes were discussed with the moderators and study staff to ensure alignment and accuracy. A narrative was then written reflecting the assigned themes and illustrated with verbatim quotes.

### Ethics

The POWWeR trial (Clinicaltrials.gov NCT05666778) obtained ethical approval for human subject's research from RUSH University in Chicago (2040505-IRB01), University of Illinois, Chicago (2023-0053), LSTM (22-076), and the Jaramogi Oginga Odinga Teaching and Referral Hospital (ISERC/JOOTRH/657/22); and has received clearance to conduct research in Kenya by NACOSTI (NACOSTI/P/24/33000) and the Kenya Pharmacy and Poisons Board (ECCT-23-07-01-2023) (40). Before starting, all participants provided written informed consent to participate in the FGDs, separate from the main study consent, and consent for audio recording. No participants were minors requiring parent consent to participate. All participants aged under 18 years met criteria for emancipated minor status under Kenyan ethical guidelines and were able to provide written informed consent to participate.

## Results

Seven FGDs were conducted between June 6th and September 15th, 2023, with each session involving 7–10 participants, totalling 57 FSWs. Participants were a median age of 27 and predominantly Kenyan (98%) from the Luo ethnic group (95%). FGD participants had engaged in sex work for a median of 4 years, with 50% of women engaged in other employment in addition to sex work. Focus group and participant characteristics are summarised in Table 1.

**Table 1:** Focus group and participant characteristics.

Focus group characteristics	Number of participants, N = 57	Duration
FGD 1	8	1 h 24 min
FGD 2	8	1 h 41 min
FGD 3	7	1 h 20 min
FGD 4	8	1 h 18 min
FGD 5	10	1 h 37 min
FGD 6	8	1 h 26 min
FGD 7	8	1 h 47 min

Participant characteristics	*N* (%)	
Median age (range)	27 (17–35)	
Birth Country	56 (98%) Kenya	
	1 (2%) Uganda	
Ethnic group/tribe	54 (95%) Luo	
	1 (2%) Kisii	
	1 (2%) Luhya	
	1 (2%) Kiswahili	
Highest level of school completed	10 (19%) Never completed school	
	26 (46%) Completed primary school	
	10 (17%) Completed secondary school	
	11 (19%) Completed vocational school, college/university	
Has any live-born children	53 (93%)	
Median number of live-born children among those with children (range)	2 (1-5)	
Has a non-paying boyfriend or husband	35 (61%) Yes	
Has other regular income outside of sex work	29 (51%) Yes	
Median years working as a sex worker (range)	4 (1–17)	
Usual locations of sex with clients (not mutually exclusive)	16 (28%) Sex den/brothel	
	46 (81%) Room at bar/club/restaurant	
	46 (81%) Lodge/hotel/guest house	
	12 (21%) Client's home	
	7 (12%) Other	
	2 (4%) Participants home	
	3 (5%) Online/mobile	
	1 (2%) Estate	
	1 (2%) Market	

### Themes

The three primary domains covered by the FGD topic guides were: (1) Current MHM practices, (2) Impact of menstruation on work and livelihood, and (3) Clients’ perceptions of sex during menses.

#### Current MHM practices

When describing current methods used to manage menses, FSWs described practices outside of sex work and during sex work. Outside of sex work, the women reported using disposable sanitary pads. Less commonly, women discussed using tampons, cloths, cotton wool, pantyliners, reusable sanitary pads, and tissue to absorb menstrual flow.

The FSWs expressed preference for using disposable sanitary pads to other absorbents due to their familiarity, easy accessibility, and reliability in preventing leaks and staying securely in place. A few also mentioned that pads were comfortable and affordable. However, many reported using disposable pads when they could afford them and switching to cloths when they couldn't.

“I have not seen any problem with it (always sanitary pad), and then again, it is cheap you can easily find it in very few minutes in the shops closer to you.” (FGD2 R7)

“… sanitary is the best to use just that the level of affordability differs, and that's what can lead us to use the piece of cloth which is not safe.” (FGD1 R7)

Some FSWs reported that discomfort from disposable pads drives them to seek alternatives. A few FSWs also expressed a preference for tampons but found them to be too expensive.

“R7: You can use it (disposable sanitary pad), and it makes sour on your thighs such that it hurts when you walk because of the friction, it makes you not to have peace as you walk forcing you to just sit still, so sometimes when I feel like my whatever (menstrual flow) has reduced, I can take cotton wool…

M: Okay, and given an opportunity, which one can you choose to use that you will be comfortable with…

R7: It is just that O.B. (tampon) is expensive, but that is what I would have wished to use, I insert it, and when it is full, I remove it.” (FGD7 R7)

When describing menstrual practices used while engaging in sex work, FSWs commonly reported intravaginal insertion of cotton or tissue to conceal bleeding. A few mentioned the need to use a lubricant when doing this.

“…because you may have children at home and you don't have money and you are in need, so it will force you to take cotton wool and insert inside your vagina to block the blood when you have sex with the client he cannot notice the blood. When you have inserted the cotton wool…you know men are different you may find that when you are having sex with a client, you feel very uncomfortable, that cotton wool you endure because it is money you are looking for, so that is the problem we are having.” (FGD4 R6)

“…once you have told him that you are on your periods, he gets so angry and he goes forever. So, once you have known that when he calls you, you will be forced to take cotton wool and insert it in there and go, because if you don't he will be upset and go forever, maybe he is one of your good clients, so you will be forced to just go, once you have inserted that thing…” (FGD7 R7)

Other methods used to continue sex work while menstruating included taking medication to delay menstruation and rinsing inside the vagina with water to temporarily stop the bleeding and allow for sex.

“…sometimes your client has come all the way from Nairobi and has really missed you and you are on your menstrual period and you know very clearly that you want to miss it (menstrual period) because he always gives you a substantial amount of money. It will force you to visit the chemist. There is a drug being bought at twenty shillings, you will take it so that it can stop the bleeding at the time that you are going to meet your client…” (FGD1 R6)

“If I go with a client and my periods are not in excess, I pass by the toilet and the cold water usually scares the blood away for approximately 10 min. Then when I get another one (client) I do the same thing with cold water.” (FGD3 R3)

However, a few FSWs reported either only engaging in sex work when their menstrual flow was light, pretending to be unaware of their period or claiming it had just started.

“Personally, when I am on my periods I pretend that I didn't know but once he has penetrated he, he has to give me my money.” (FGD5 R3)

Others informed the client and proceeded with sex as usual if the client was agreeable.

“For me, that (intravaginal insertion of cotton) is something I have never done. What I know is that I talk to the client and tell them that I’m in my periods, so if he is understanding then we can even push it forward, because it can be dangerous inserting something.” (FGD2 R6)

When describing their hygiene practices, a few FSWs maintained their usual bathing or showering routine during menstruation, but more often, FSWs reported increasing the frequency of bathing. A few FSWs reported that increased bathing was necessary to prevent bad odours. FSWs also reported half-bathing or wiping their genitals, particularly when changing sanitary pads, while a few preferred to take a full bath at these times. A small number mentioned avoiding soap when washing their genitals. Frequently changing sanitary pads was a common practice cited to prevent bad odours.

“… when in your menses you have to bath three times … you have to maintain cleanliness, and then you should also be changing the pads, because if you do not, during menses you tend to have some smell, you will not be able to go closer to people, you have to maintain cleanliness because cleanliness is next to Godliness.” (FGD2 R7)

Additionally, a few FSWs stated that the risk of infection could increase during menses if reusable cloths were not properly washed, dried, and stored.

“… they have to maintain the patch cloth by ensuring they are clean by washing them and drying them using sunlight you don't leave them in shade for drying since there are the small flies that when they get into contact with the cloth and when you again use the cloth on the private part it can lead to an infection. so, we do keep those stuff of ours very clean as we use them.” (FGD1 R1)

#### Impact of menstruation on work practices and livelihood

There was roughly an even divide between those who do and those who do not engage in sex work during this time. Few FSWs expressed a positive attitude toward having sex during menstruation; for most, it was seen as a financial necessity rather than a preference. However, a small number reported a higher libido during menstruation.

“In sex work there is time the business is low, there is a time you don't have money and the children also need to feed, it will force you to go and have sex.” (FGD4 R)

Reasons for avoiding sex work while menstruating included pain or discomfort during sex, fears of contracting or transmitting diseases/infections, menstrual symptoms (backache, lethargy, cramps, bad moods), low libido and beliefs that it is unacceptable. Additionally, a few FSWs described wanting to let the menstrual blood flow out uninterrupted to avoid stomach pains.

“I do not go because I am not comfortable, yes, I am not comfortable, just thinking that someone wants to insert his thing in the blood, I am very shy I cannot spread my legs.” (FGD7 R5)

“Okay, when you have sex when you on your period, I hear you can get a pelvic inflammatory disease and this disease…you will experience back pains and pain on your lower tummy when you have sex during periods…” (FGD3 R2)

“Personally it is only sex that I do not have when menstruating, because when I do the lower part of my belly kills me and the flow is a lot…” (FGD2 R4)

The most commonly reported impact of menstruation on participants' livelihoods was a loss of income due to either stopping work or reducing their number of clients. A few FSWs noted changes in their clientele, such as choosing to see only familiar clients or those who pay well.

“When I’m in my menses, because now I will not be doing most of the work, I do not go to the ‘field’. I stop doing a lot of work that involves getting money…” (FGD2 R4)

“When I am on my (menses) I mostly go with my regular clients who I am used to. There are some clients who come and wants you to tell stories first for a little bit. Sometimes it depends on the money that he is giving you, because even as you sit, you know that I will get this amount of money…” (FGD5 R1)

Among those who stopped sex work during menstruation, the FSWs often reported taking up a second job, receiving financial support from a regular client, or relying on their savings.

“… you will have to rise up and go search to find [a job] even if it is ‘washing’ just for you to find something to eat because now the situation has proved to be difficult.” (FGD1 R2)

“I will just tell him that just come but on the enjoyment part we will have to forego because am in a bad situation and you know there are those (clients) who are really good… I have one of my clients who when he comes when am in that situation (menstruating), he tells me that just come so long as I get to see you and, truthfully, he sees me and when he leaves he gives me a substantial amount of money to get home with. He knows how my child goes to school, he knows how I eat and even takes care of rent…” (FGD1 R1)

For those who continued sex work while menstruating, the most commonly reported difficulties with continuing work included the pain and discomfort of inserting and removing cotton or tissue, and the discomfort of having these materials in the vagina during sex. The FSWs described that cotton and tissue could become lodged or lost in the vagina, with tissue splitting into many pieces. The women also reported these materials can cause a foul smell, especially if left in for a while.

“…be with one client finish with him and then place another one (tissue into the vagina) go to the next client and remove again gets to a point you start feeling pain down there that is the first challenge. The second one, when you insert the first time and use it for some time, let us say three hours, then you see like blood has started to whatever again and remove it, and when you are removing it, it breaks into tiny pieces.” (FGD5 R9)

“…so when you want to change, you look for it (the inserted cotton wool) and you don't get it … At times it is stressful it has disappeared and you go to the hospital the next day and you feel uncomfortable because it has over stayed and you start smelling inside there.” (FGD4 R4)

A few also described sex during menstruation as generally painful or uncomfortable and mentioned issues such as bad moods, tiredness, low libido, and concerns about staining the sheets.

“… I shouldn't have sex, usually it makes me tired when I am menstruating. You become moody, you are in a bad mood, so like we as sex workers we are forced to go and have sex…” (FGD5 R2)

#### Clients' perceptions of sex during menses

While a few FSWs reported that some clients were completely unaware of menstruation, the majority acknowledged that men generally know that women menstruate. However, FSWs noted that some men's understanding of menstruation was poor, with some men believing that during menstruation, women are more fertile or at higher risk of transmitting diseases.

“Okay, they know but what is stuck in their minds is pregnancy…you can get a client who wants to have an intercourse with you without a protection. Client will ask you ‘are you on your periods?’ or ‘Will you tomorrow come complaining that you are pregnant?’, so what is on their mindset is only pregnancy.” (FGD3 R5)

Married and elderly men were often perceived as the most knowledgeable and understanding, often gaining their knowledge from their wives. These men were more likely to provide financial support during menstruation without engaging in sex. In contrast, a number of FSWs reported that younger men were less knowledgeable and understanding and more likely to be insistent on sex. None of the FSWs noted differences in knowledge or understanding based on a client's educational level or employment status.

“… the youths I can say that they do have the mind of children, he will not mind that I attend, him he does not know; but the old ones, you tell him that you are in this state (menstruating), he will tell you that he knows that problem of women. Some will also tell you that having sex with someone on menses will bring lower stomach pain to the lady or that the lady can give him (the pain), and when you are on period and have sex then that will bring that problem… so I feel that the older people have more information than the young.” (FGD6 R1)

The FSWs reported a range of client attitudes towards sex during menstruation, from indifference to disgust, with most observing that men generally held negative views. Some FSWs reported that men were afraid of menstrual blood and infections, and a few noted that they may complain about the wetness.

“The older clients if you go and have sex with them and they see you removing the pad he will tell you ‘aah-aah don't hide it just take the money, I cannot have sex with you when you are in your menses.’ But the young men will not leave it even if you are having your menses, even if it is coming out like pus” (FGD4 R1)

“What he said is that he is afraid of contacting a disease, not this story of being surprised by sex. What they always fear is that you can be positive or have infected him.” (FGD3 R3)

“The private part has to be tight. If it is not tight and is loose, yourself you lose the confidence because you are ridiculed that you are watery, that ‘today I got one who is so abnormally watery’…” (FGD1 R7)

While very few FSWs reported hearing of or encountering men who preferred to have sex with menstruating women, those who did mentioned that these men might offer more money or believe that women are “sweet” or “hot” during their period.

“There are those who love it … You had told him 300, if you tell him you are in your periods he will leave you with 500 he can add you even 100… It is thick (sweet)… Some feel like licking that blood” (FGD4 R)

Some FSWs reported that occasionally, clients did not believe they were menstruating and asked for proof. Other FSWs mentioned that if they themselves were surprised by their periods, clients could be sceptical about the timing.

“But there are some who do not listen. Because you can find that he has sex even with the wife who is in the house when they are menstruating. It is not someone who is understanding, but there are good ones whom when you tell they tell you to just be and call them when finish when you finish…you realize that actually you are just doing well with that client, but the kind that refuse to listen, it gets to a time that you yourself does not know what to tell them…you avoid them, because you want to protect yourself, because when he has sex with you when you are in your menses, you will have to feel some pain below your belly.” (FGD2 R1)

Many reported conflicts with clients who became angry or upset, with some questioning why they weren't informed about menstruation beforehand. FSWs commonly reported that clients would either refuse to pay or reduce the agreed-upon amount. However, several FSWs noted that they require payment from the client before any sexual activity. While a few FSWs stated they had not experienced or heard of physical abuse from clients upon discovering menstruation, several reported such incidents.

“Some people when you go with them and seeing the blood they get discouraged. They say like ‘you are on periods and you can't tell me, you only wait until you are here and then it's me who realizes it, then there is nothing that we can do.’ He tells you that just leave even without escorting you or giving you any money, he leaves just like that.” (FGD3 R2)

“I went with a client little did I know that the tissue I had inserted had absorbed a lot of blood while I thought that I was safe. After he had finished and on seeing that the condom has blood, this man was ready to beat me up. I had to return the money by that time I wasn't booking a room, we were using the one that you pay for the room when you are leaving, I was forced to pay for the room with my own money.” (FGD5 R9)

“We had agreed on 2k, I was almost strangled for that 2k, I didn't believe it…He noticed (blood) and when he did he said ‘you will give me, you will give me my money.’ He held me like this [gestures] and told me ‘I don't f*** a woman who is on her periods.’ He was really angry. I was held by the neck and thrown to the other side, and opened the door and left, what could I have done?” (FGD5 R2)

The majority of FSW accounts reported keeping menstruation secret from clients, using the above-mentioned methods to conceal it, or pretending that it had just started. This was driven by fears that clients would refuse sex if they knew. A few FSWs said their decision to inform clients depended on their financial situation.

“You can keep quiet about it according to the level of need you have for money. Sometimes he shows you the money, yet your children are sleeping without a meal, and you have no means of how to assist them… you will have to keep quiet about it.” (FGD1 R1)

Among the few who chose to inform clients, some did so only with familiar ones, believing they are more understanding. However, a few FSWs reported informing only new clients, as they felt more comfortable managing their familiar clients’ reactions.

“I think it is good to tell them so that if he can continue…he can go ahead but if he cannot, then he can just go.” (FGD2 R2)

## Discussion

This research sought to describe FSWs' accounts of their everyday practices in managing menstruation and its impact on their work practices, livelihoods and client relations using qualitative FGDs. Our findings highlight the common use of potentially harmful materials and practices to conceal menstrual blood during sex work, underscoring the need for improved MHM products to support FSW's menstrual needs during work. Our results show how negative perceptions of menstruation, held by both FSWs and their clients, contribute to reduced work opportunities, and increased financial instability and risk of violence.

Similar to other studies (31, 47, 48) the majority of FSWs reported using sanitary pads to manage menstrual flow outside of sex work. Women reported sores and irritation from disposable pads and faced difficulties with the high cost of tampons and pads, adding to an extensive body of literature on the poor quality and unaffordability of appropriate menstrual products in LMICs (27). In 2015, it was reported that 65% of Kenyan women and girls could not afford sufficient products as the price of a package of disposable sanitary pads equalled the daily wages of an unskilled worker (49). In LMICs, managing reusable menstrual products is difficult due to limited access to soap, water, privacy, and adequate facilities, with washing and carrying used products often viewed as unpleasant and inconvenient (50). In Kenya, only 41% of the population has access to at least basic sanitation services, and 54% of the household population do not have drinking water on their premises (51). Menstrual taboos discourage open washing and drying, often resulting in damp cloths being worn, posing health risks (27, 52). These commonly raised challenges among participants underscores the need for alternatives that can alleviate both the discomfort and financial burden associated with current menstrual products for many FSWs.

Participants generally expressed negative attitudes towards engaging in sex work during menstruation. FSWs in our study reported needing to continue taking clients during this time due to economic hardship, echoing findings across other studies in this population (8–10, 12). Most FSWs, an already economically constrained population, experienced loss of income due to stopping work or reducing their number of clients. Similar to working women in many other job sectors (53), interventions are needed to ensure that menstruation does not negatively impact economic stability, with attention to the unique needs of having sex during menses. It is for this reason that we are trialling a style of menstrual cup that can be worn during sex (40, 54).

We found that the majority of FSW's accounts described keeping menstruation a secret, fearing clients would refuse sex if they were aware. To conceal menstruation, many women resorted to potentially harmful practices to hide blood flow, including intravaginal cleansing and inserting cotton or tissue into the vagina, consistent with the limited literature available (8–10, 12, 13). FSWs indicated that insertion of these materials often resulted in pain and discomfort, items becoming lost or stuck in the vagina, and bad odours. This aligns with findings from the study of FSWs in Brazil, where 42% used “cotton and other substances” to hide menstruation (10). Additionally, a number of women in our study reported pretending their menses had just started or using medication to temporarily stop their menses in order to continue sex work. This aligns with Chacham and colleagues' (2007) study, which reported the continuous use of hormonal contraceptives among FSWs to suppress menses (10). Our findings reveal that financial need and negative client attitudes towards sex during menstruation significantly impact participants' work practices, creating pressure to conceal menstruation through potentially harmful methods.

The majority of FSWs believed that men are generally aware that women menstruate monthly but that they lack in-depth knowledge, in keeping with the growing body of research highlighting men's lack of information regarding menstruation (55–60). A few FSWs reported that some men believed women are more fertile or more likely to transmit diseases during their menses. This was echoed by Allen and colleagues (2010) (9) and in our FGDs with male clients of FSWs in Kisumu, who expressed fears of contracting “Pelvic Inflammatory Disease” or “dangerous” STIs from “dirty” menstrual blood (15). A study using in-depth interviews with 15 fathers in rural western Kenya found that most believed women could become pregnant during menstruation and avoided sex during this time (60). These misconceptions may fuel stigma, reinforce harmful stereotypes, and contribute to use of dangerous practices to conceal menstrual blood.

FSWs reported a range of client attitudes towards sex during menstruation, from indifference to disgust, with most observing that men generally held negative views. This was echoed in our FGDs with male clients of FSWs, who described sex during this time as forbidden, risky, or unpleasurable. Some men specifically sought services from FSWs because their usual sex partner (e.g., girlfriend, wife) was menstruating (15). Male customers of women working in food and recreation facilities in Tanzania reported that sex during menstruation was generally avoided, except when they had not had sex for a long time or were drunk. However, the study also found that bar and guesthouse workers attributed many cases of sex during menstruation to male coercion and fears of losing male partners (9). While very few FSWs reported hearing of or encountering men who preferred to have sex with menstruating women, those who did mentioned that these men might offer more money or believe that women are “sweet” or “hot” during their period. This is supported by MacLean and colleagues (2020) study among secondary school-aged girls in Oyugis, Kenya, where some participants reported that men pursued them most when they were menstruating (61). Additionally, male participants in MacLeod and colleagues' study (2023) described using self-control to avoid sex during menstruation rather than lacking a desire for it (62). Improving clients' understanding of menstruation could promote more supportive attitudes and create a safer, more respectful work environment for FSWs.

For FSWs who did not want to continue sex work during menstruation, some reported that clients were understanding and agreed to wait until menstruation ended, while others insisted on sex regardless. Those who continued sex work during menstruation reported clients frequently refused services if aware of menstruation, often becoming angry or upset, and women reported instances of physical abuse from clients upon discovering menstruation. These narratives align with our FGDs conducted with male clients that found men felt deceived upon realising a FSW was menstruating but proceeded with sex to relieve their sexual urges. Conversely, a few reported they might ask for their money back and leave without having sex (15).

These findings directly informed the implementation approach of the menstrual disc intervention within the POWWeR trial to ensure that the intervention was responsive to the lived realities of FSWs. The need to discreetly manage menstruation during sex, alongside widespread reliance on harmful intravaginal practices to conceal bleeding, highlighted the importance of positioning the menstrual disc as a method that can be used during intercourse while reducing the need for insertion of unsafe materials or frequent vaginal cleansing. These insights informed intervention training content, including practical guidance on managing client interactions.

## Limitations

These FSW narratives may have been influenced by desirability bias; however, there was a strong level of congruence between responses across FGDs which suggests that we captured the key participant perspectives. Participants in this study were recruited through peer champions and community networks and may not be fully generalizable to the broader population of FSWs in western Kenya. The coding of the transcripts was completed by a single researcher who was not involved in data collection and unfamiliar with Kenyan culture, which may have limited understanding of nuances. Measures taken to mitigate the risk of unconscious bias and misinterpretation included keeping a reflective diary to identify any biases and reviewing the draft alongside the original transcripts to ensure the context and significance of the findings were not misinterpreted or overstated. Support and input from the research study team were included. The lead authors and project coordinator attended FGD debriefs, read and re-read the transcripts to ensure familiarity with the data, discussed and agreed on the final coding frame and agreed on the overall themes.

## Conclusion

This study adds to the limited research on FSWs' menstrual hygiene practices. The research depicts the commonness of potentially harmful practices, such as intravaginal cleansing and insertion of cotton to conceal menstrual blood during sex, and the reasons underlying these practices. It underscores the critical need for safer menstrual management solutions that can be used discreetly and comfortably during sex, such as menstrual discs that can be worn during sex. The study also reveals how menstruation may contribute to the economic precarity of FSWs due to their own and clients' misconceptions and negative perceptions, which contribute to reduced work, clients' refusal to pay, and increased risk of violence. This highlights the need to also educate men to reduce menstrual stigma and promote safer sexual interactions.

## Data Availability

The datasets presented in this study can be found in online repositories. The names of the repository/repositories and accession number(s) can be found below: https://doi.org/10.25417/uic.28789268.v1.
