# Supplementary material for: A qualitative exploration of menstrual management practices and the impact of menstruation on the livelihoods of female sex workers in a peri-urban area of western Kenya
Source: Front Reprod Health. 2026 Jun 5;8:1817624. doi: 10.3389/frph.2026.1817624 (PMC13279765; doi:10.3389/frph.2026.1817624)
Supplement: Supplementary file 1 [file Table1.docx]

| **Topic:** | **Probes:** |
| --- | --- |
| **Overview/ Warm up: meaning of menstruation:** | What does menstruation mean to you? Is there anything good about having a period? What / why? Is there anything bad about having a period/ what / why? Is there anything you should or should not do while you are having your period? Why? How do you feel about this? |
| **Current absorbent** | What do you use to absorb your menstrual flow? Why do you use this? What are the good things about it? Are there any disadvantages to using this? (infections/cost/obtaining/disposing) Do you have any difficulties in using this? (access, cost, WASH) If you were able to choose something else – would you? What would this be? Why? |
| **Hygiene practices** | How do you keep clean during your menses? Is this routine different to your cleaning routine when you are not having your period? In what way? How difficult is this to do (normally vs during menses)? (access, cost, WASH) |
| **Impact on livelihood** | Do women like yourselves work when you are having your menses? Why? / Why not? Is it difficult? Why / why not? How does this impact on your livelihood? Do you have a preference about working when you are having your menses? Can you explain your answer. |
| **Client knowledge and views of menses** | In general what do your clients know about menstruation? Does this differ across groups, e.g. age, education, marital status. What are their views about menstruation? Does this differ across groups, e.g. age, education, marital status |
| **Client perceptions of sex during menses** | What about having sex during menstruation, how do your clients feel about this? Do they change their behaviours (why / how?). Do you need to keep your menses hidden from your clients? Why? Do you agree with this? Does this differ by type of client? (e.g., regular vs. new client, age, education, marital status) What do you do to try and achieve this? How successful is it? Are there any problems in doing this? (infections / cost / supplies / client reaction) How do you feel about doing this? Do you ever hear about bad experiences if a client has found out a woman was having her menses? What happens / happened? Do clients ever prefer a woman to be menstruating during sex? Why? |
| **Initial thoughts on menstrual cup** | Have you heard of the menstrual cup? What do you know about it? What are your thoughts about it? What might be good? What might be bad? How does menstrual cup compare to your current way of managing your menses? (e.g., advantages, disadvantages) |
| **Closure:** | Is there anything else you think it is important to tell us about having your menses? What about working during your menses? What about your clients? What are your final thoughts about the menstrual cup? |

**Additional File 1: Focus Group Discussion Guide on Menstrual Health Practices among Female Sex Workers**
